# The Response of the Functional Traits of *Phragmites australis* and *Bolboschoenus planiculmis* to Water and Saline–Alkaline Stresses

**DOI:** 10.3390/plants14142112

**Published:** 2025-07-09

**Authors:** Lili Yang, Yanjing Lou, Zhanhui Tang

**Affiliations:** 1 Key Laboratory of Wetland Ecology and Vegetation Restoration, Ministry of Ecology and Environment, Key Laboratory for Vegetation Ecology, Ministry of Education, School of Environment, Northeast Normal University, Changchun 130117, China; yanglili@nenu.edu.cn; 2 Jilin Provincial Joint Key Laboratory of Changbai Mountain Wetland and Ecology, Northeast Institute of Geography and Agroecology, Chinese Academy of Sciences, Changchun 130102, China

**Keywords:** functional traits, adaptive strategies, saline–alkaline stress, water stress, salt marsh

## Abstract

Soil saline–alkaline stress and water stress, exacerbated by anthropogenic activities and climate change, are major drivers of wetland vegetation degradation, severely affecting the function of wetland ecosystems. In this study, we conducted a simulation experiment with three water levels and four saline–alkaline concentration levels as stress factors to assess eight key functional traits of *Phragmites australis* and *Bolboschoenus planiculmis*, dominant species in the salt marsh wetlands in the western region of Jilin province, China. The study aimed to evaluate how these factors influence the functional traits of *P. australis* and *B. planiculmis*. Our results showed that the leaf area, root biomass, and clonal biomass of *P. australis* significantly increased, and the leaf area of *B. planiculmis* significantly decreased under low and medium saline–alkaline concentration treatments, while the plant height, ramet number, and aboveground biomass of *P. australis* and the root biomass, clonal biomass, and clonal/belowground biomass ratio of *B. planiculmis* were significantly reduced and the ratio of belowground to aboveground biomass of *B. planiculmis* significantly increased under high saline–alkaline concentration treatment. The combination of drought conditions with medium and high saline–alkaline treatments significantly reduced leaf area, ramet number, and clonal biomass in both species. The interaction between flooding water level and medium and high saline–alkaline treatments significantly suppressed the plant height, root biomass, and aboveground biomass of both species, with the number of ramets having the greatest contribution. These findings suggest that the effects of water levels and saline–alkaline stress on the functional traits of *P. australis* and *B. planiculmis* are species-specific, and the ramet number–plant height–root biomass (RHR) strategy may serve as an adaptive mechanism for wetland clones to environmental changes. This strategy could be useful for predicting plant productivity in saline–alkaline wetlands.

## 1. Introduction

Saline–alkaline wetlands are mainly distributed in inland plains, plateaus, and basins within arid or semi-arid regions. In recent years, the increasing frequency of extreme hydrological events, alongside the rising accumulation of soil salinity due to anthropogenic disturbances and global environmental changes, has subjected wetland plants to escalating water, saline, and alkaline stresses [1,2]. These factors have profoundly affected the structure and function of wetland ecosystems [3]. A comprehensive understanding of how saline–alkaline wetland plants adapt to soil salinization, alkalization, and water stress is critical. This knowledge is essential for improving conservation strategies for these species. A comprehensive understanding of the adaptive strategies employed by saline–alkaline wetland plant species is crucial for their effective conservation. These strategies help them cope with challenges such as soil salinization, alkalization, and water stress. Moreover, it is critical for the scientific management of these fragile wetland ecosystems in the face of ongoing environmental changes.

Plant functional traits refer to a set of core characteristics closely related to plant colonization, survival, growth, and mortality [4]. These traits, either individually or in combination, reflect a plant’s response and adaptation strategies to environmental changes, thereby influencing ecosystem functioning [5]. Variations in plant functional traits are indicative of species-specific adaptation strategies and their capacity to cope with environmental conditions [6]. The phenotypic plasticity of these traits enables plants to optimize resource acquisition and enhance stress tolerance, providing a mechanistic perspective for understanding ecological trade-offs. Integrating plant functional traits with stress response analyses may provide valuable insights for the restoration and management of degraded saline–alkaline wetland ecosystems.

Saline–alkaline stresses pose significant challenges to the colonization, growth, reproduction, and distribution of wetland plants, inhibiting plant growth and, in extreme cases, leading to plant mortality [7]. Over prolonged periods of adaptation and evolution, wetland plants have developed regulatory mechanisms that are both morphologically and physiologically adapted to saline–alkaline environments. These adaptations include reductions in plant height and leaf area, adjustments in litter and root production, and trade-offs between reproduction and growth, as well as between aboveground and belowground resource allocation [8,9]. Unlike coastal salt marshes, which are primarily characterized by saline stress (i.e., NaCl), inland saline–alkaline wetlands mainly face alkaline stress (i.e., NaHCO_3_), which combines both saline and alkaline stresses [3,10]. Research has shown that combined saline and alkaline stresses inhibit wetland plant growth more severely than saline stress alone [3,11]. However, most existing studies have predominantly focused on the responses of coastal salt marsh plants to saline stress. There remains a significant gap in understanding the morphological and physiological adaptive strategies of typical wetland plants in inland saline–alkaline wetlands under combined saline and alkaline stress. Additionally, the mechanisms through which plant functional traits respond to saline and alkaline stress are not yet fully understood and require urgent investigation.

Hydrological conditions are fundamental determinants of wetland plant communities, with these ecosystems frequently exposed to fluctuating water levels [12,13]. Moisture stress, including both drought and flooding, represents a major challenge to wetland plant growth. Flooding negatively affects wetland plants by reducing gas diffusion rates and oxygen solubility in water, thereby inhibiting plant growth [14,15]. Conversely, drought can severely affect the growth and metabolism of many wetland plants [3,16,17]. Growth strategies in plants reflect the trade-off between dry matter production and nutrient conservation [18,19]. The effect of flooding on biomass allocation to different plant organs has been confirmed in several studies, highlighting adaptations such as the formation of aerenchyma and adventitious roots, increased investment in belowground resources, and enhanced clonal propagation [20,21]. In saline–alkaline wetlands, inundation accompanied by saline–alkaline conditions represents a common dual stressor, but much of the existing literature primarily focuses on the impacts of water level or saline stress in isolation, with fewer studies addressing the interactions between these two stressors.

The western region of Jilin province is a representative area for the development of inland marsh wetlands. However, soil saline–alkaline stress, combined with increasing extreme flooding and drought events driven by global changes and human activities, has significantly degraded the marsh wetlands in this region. Consequently, the area of these wetlands has reduced from approximately 6.4 × 10^4^ km^2^ in 1954 to 1.6 × 10^4^ km^2^ in 2008 [3,22]. *P. australis* and *B. planiculmis* are the dominant species in the marsh wetlands of the western region of Jilin province. *P. australis* is a tall, perennial grass characterized by a robust and extensive rhizome system, making it one of the most widely distributed and productive plant species globally [23]. *B. planiculmis*, a perennial herb from the Salicaceae family, produces bulbs that serve as a major food source for the critically endangered whooping crane (*Grus leucogeranus*) [24]. Both *P. australis* and *B. planiculmis* are perennial species with extensive rhizome or bulb systems and exhibit a degree of salinity–alkalinity tolerance, making them ideal candidates for studying wetland plant functional traits and their responses to environmental changes [25]. Despite the growing interest in salt marsh vegetation dynamics in recent decades, there remains a scarcity of studies addressing the adaptive strategies of dominant salt marsh plants, such as *P. australis* and *B. planiculmis*, in response to water stress and saline–alkaline stress.

Plant functional traits, which encompass various morphological and physiological characteristics, are key indicators of a plant’s ecological strategies in adapting to extreme environmental factors [26,27]. Numerous studies have explored the relationship between plant traits, ecosystem properties, and services, but studies focused on saline–alkaline wetlands remain limited. Furthermore, an integrated analysis of environmental gradients, plant traits, and ecosystem properties is crucial for understanding their interactions and responses [28,29]. This study aims to investigate the response of functional traits in *P. australis* and *B. planiculmis* during the germination period to varying saline–alkaline concentrations and water levels under controlled conditions. The primary objectives of this study are as follows: (1) to assess the response of functional traits of *P. australis* and *B. planiculmis* to various saline–alkaline concentrations, (2) to examine the changes in functional traits of these two species in response to varying water levels under saline–alkaline conditions, and (3) to evaluate the effects of water and saline–alkaline stresses on key functional traits of *P. australis* and *B. planiculmis*, as well as the predictive capacity of these traits for plant production performance.

## 2. Results

### 2.1. Relationships Between Key Functional Traits and Other Functional Traits in P. australis and B. planiculmis

**Figure 1:**
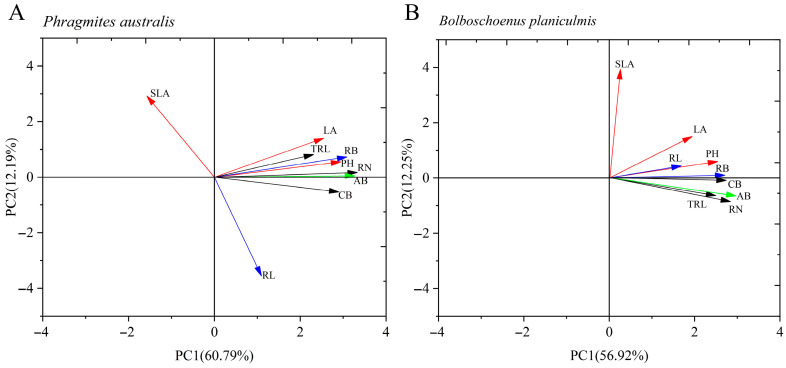
Two-dimensional PCA ordination plot of functional traits for *P. australis* and *B. planiculmis*. (**A**) *P. australis* and (**B**) *B. planiculmis*. PH: Plant height; LA: Leaf area; SLA: Specific leaf area; RL: Root length; RB: Root biomass; RN: Ramet number; TRL: Total rhizome length; CB: Clonal biomass; AB: Aboveground biomass. A total of 120 pots were used in this experiment, containing 60 *P. australis* and 60 *B. planiculmis* plants, with one plant per pot.

**Figure 2:**
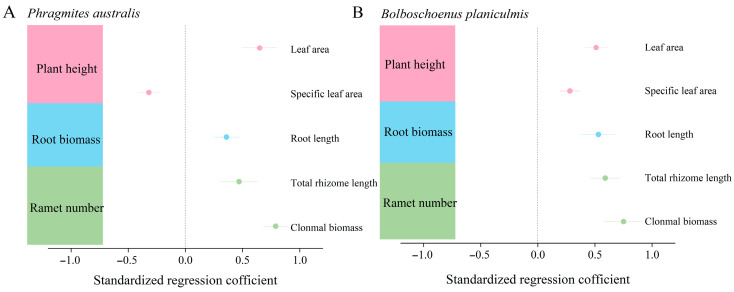
Linkages between plant height, ramet number, and root biomass with plant functional traits in *P. australis* and *B. planiculmis*. (**A**) *P. australis* and (**B**) *B. planiculmis*. All parameters displayed significant effects on plant resource acquisition (*p* < 0.05). Data points indicate the standardized regression coefficients obtained through linear regression analysis, while the lines indicate the 95% confidence intervals. A total of 120 pots were used in this experiment, with 60 *P. australis* and 60 *B. planiculmis* plants, one plant per pot.

Principal component analysis (PCA) revealed that the total variance explained for *P. australis* was 72.98%, with the first principal component (PC1) contributing 60.79% and the second principal component (PC2) contributing 12.19%. For *B. planiculmis*, the total variance explained was 69.17%, with 56.92% from PC1 and 12.25% from PC2 (Figure 1A,B). The key functional traits of both species were primarily determined by the first principal component, which was mainly explained by plant height, ramet number, and root biomass. For light acquisition traits, in *P. australis*, leaf area was significantly and positively correlated with plant height and significantly and negatively correlated with specific leaf area (*p* < 0.05; Figure 2A). In *B. planiculmis*, plant height was significantly and positively correlated with leaf area (*p* < 0.05; Figure 2B). For nutrient and water acquisition traits, root biomass showed a significant positive correlation with root length in both *P. australis* and *B. planiculmis* (*p* < 0.05; Figure 2A,B). Regarding clonal reproduction traits, ramet number was significantly and positively correlated with total root length and clonal biomass in both *P. australis* and *B. planiculmis* (*p* < 0.05; Figure 2A,B).

### 2.2. Response of Functional Traits of P. australis and B. planiculmis to Saline–Alkaline Concentration

**Figure 3:**
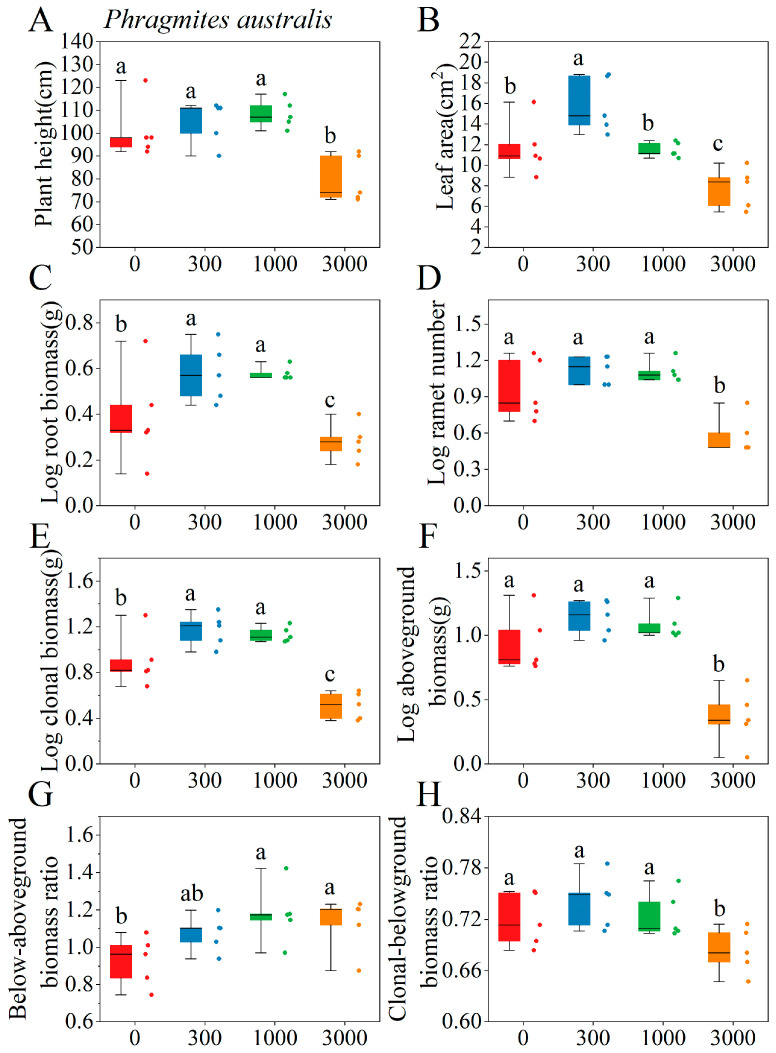
Effects of different saline–alkaline concentrations on the functional traits of *P. australis*. (**A**) Plant height; (**B**) leaf area; (**C**) root biomass; (**D**) ramet number; (**E**) aboveground biomass; (**F**) clonal biomass; (**G**) below/aboveground biomass ratio; (**H**) clonal/belowground biomass ratio. The experiment included 20 pots, each containing one *P. australis* plant, for a total of 20 plants. Different lowercase letters indicate significant differences in functional traits across varying saline–alkaline concentrations (*p* < 0.05), based on Tukey’s post hoc tests.

**Figure 4:**
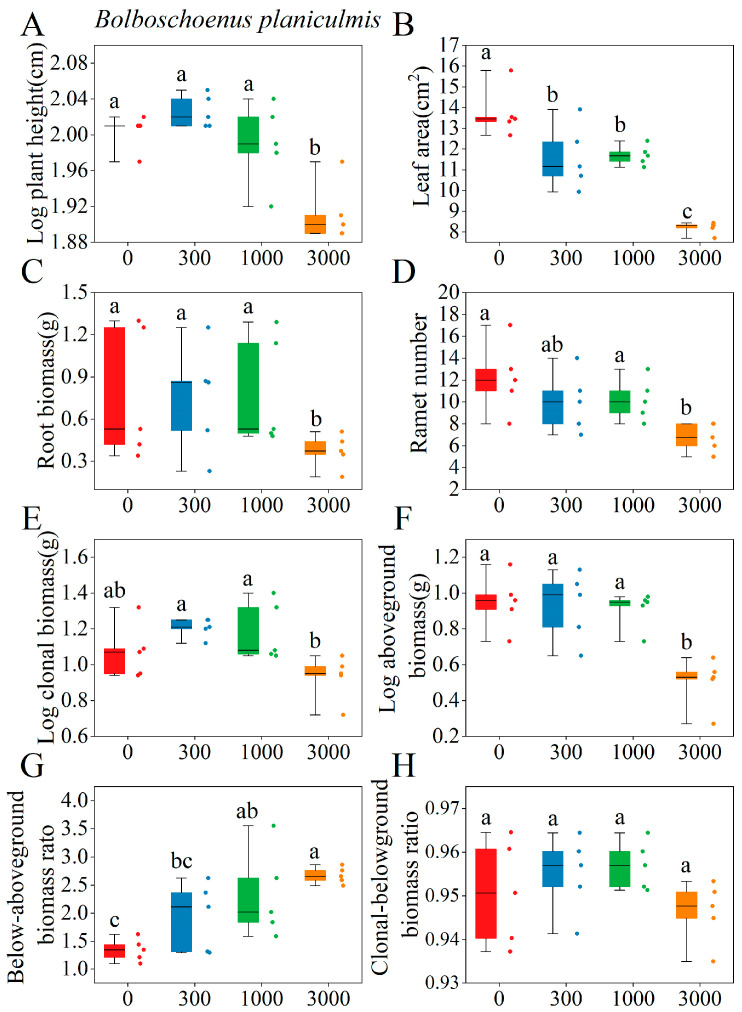
Effects of different saline–alkaline concentrations on the functional traits of *B. planiculmis*. (**A**) Plant height; (**B**) leaf area; (**C**) root biomass; (**D**) ramet number; (**E**) above-ground biomass; (**F**) clonal biomass; (**G**) below/aboveground biomass ratio, (**H**) clonal/belowground biomass ratio. The experiment included 20 pots, each containing one *B. planiculmis* plant, for a total of 20 plants. Different lowercase letters indicate significant differences in functional traits across varying saline–alkaline concentrations (*p* < 0.05), based on Tukey’s post hoc tests.

The effects of saline–alkaline concentration on the key functional traits of *P. australis* and *B. planiculmis* varied depending on species and corresponding traits (Table S1). For *P. australis*, saline–alkaline stress significantly affected functional traits such as leaf area, ramet number, root biomass, and aboveground biomass (*p* < 0.05), with the exception of root length (Table S1). Low-to-medium saline–alkaline concentration treatments significantly increased leaf area, root biomass, and clonal biomass (Figure 3B,C,E), while high saline–alkaline concentration treatment significantly reduced plant height, ramet number, and aboveground biomass (Figure 3A,D,F). For *B. planiculmis*, saline–alkaline stresses significantly affected plant height, ramet number, root biomass, and aboveground biomass (*p* < 0.05), with the exception of specific leaf area and total rhizome length (Table S1). All of the saline–alkaline concentration treatments significantly reduced the leaf area (Figure 4A), while medium-to-high saline–alkaline concentration treatments significantly increased the below/aboveground biomass ratio (Figure 4G). High saline–alkaline concentration treatment significantly decreased root biomass, clonal biomass, and the clonal/belowground biomass ratio (Figure 4C,E,H).

### 2.3. Water Level Induced Changes in the Functional Traits of P. australis and B. planiculmis in Response to Saline–Alkaline Concentration

**Figure 5:**
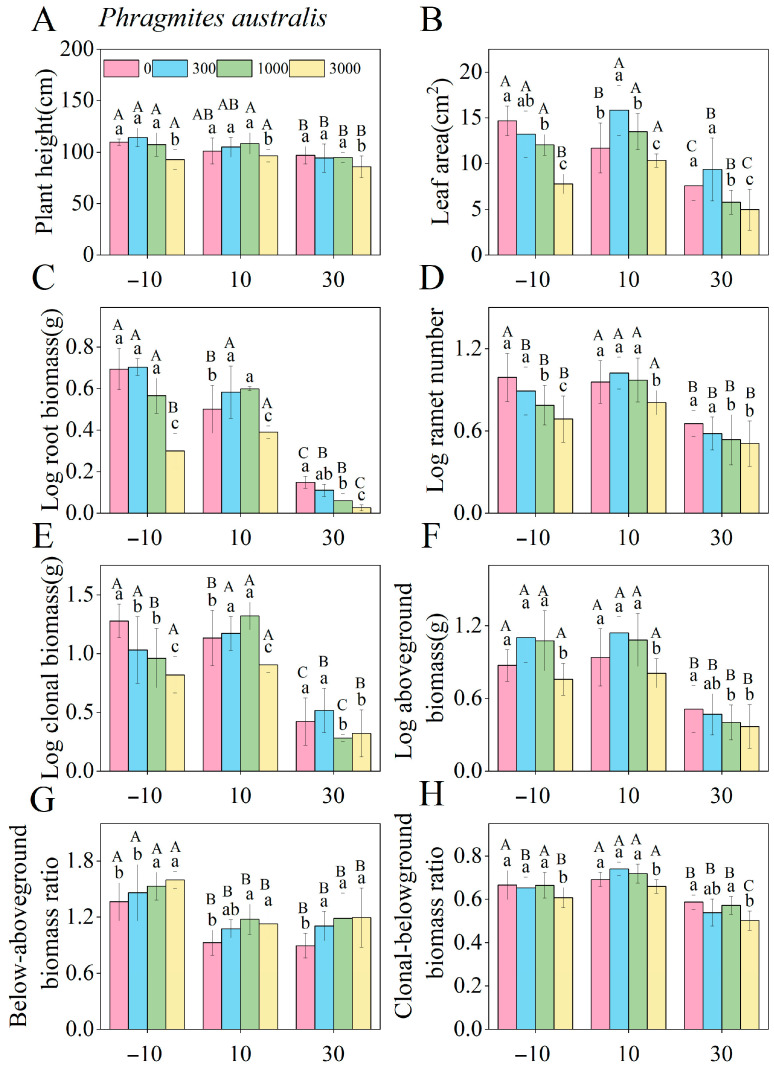
Combined effects of different water levels and saline–alkaline concentrations on the functional traits of *P. australis*. (**A**) Plant height; (**B**) leaf area; (**C**) root biomass; (**D**) ramet number; (**E**) above-ground biomass; (**F**) clonal biomass; (**G**) below/aboveground biomass ratio; (**H**) clonal/belowground biomass ratio. The experiment included 60 pots, each containing one *P. australis* plant, for a total of 60 plants. Different lowercase letters indicate significant differences (*p* < 0.05) among saline–alkaline concentrations at the same water level, based on Tukey’s post hoc tests, while different uppercase letters indicate significant differences (*p* < 0.05) among water levels at the same saline–alkaline concentration, also based on Tukey’s post hoc tests. A total of 120 pots were used in this experiment, with 60 *P. australis* and 60 *B. planiculmis* plants, one per pot.

**Figure 6:**
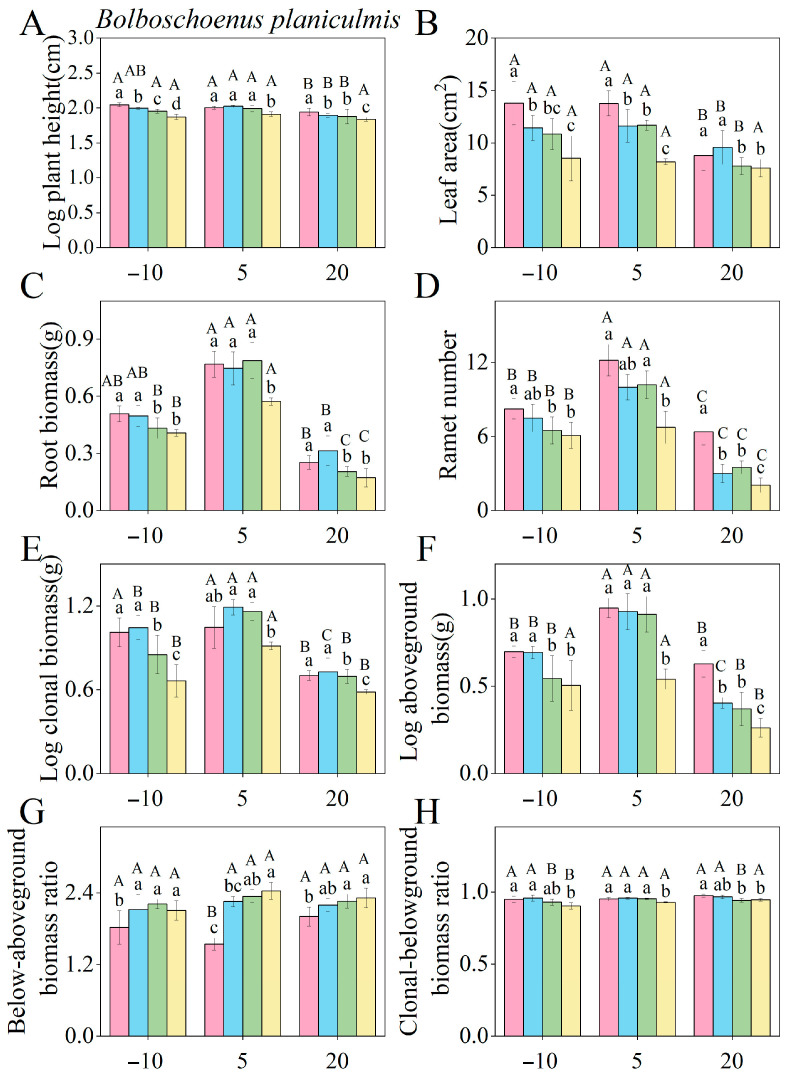
Combined effects of different water levels and saline–alkaline concentrations on the functional traits of *B. planiculmis*.(**A**) Plant height; (**B**) leaf area; (**C**) root biomass; (**D**) ramet number; (**E**) above-ground biomass; (**F**) clonal biomass; (**G**) below/aboveground biomass ratio; (**H**) clonal/belowground biomass ratio. The experiment included 60 pots, each containing one *B. planiculmis* plant, for a total of 60 plants. Different lowercase letters indicate significant differences (*p* < 0.05) among saline–alkaline concentrations at the same water level, based on Tukey’s post hoc tests, while different uppercase letters indicate significant differences (*p* < 0.05) among water levels at the same saline–alkaline concentration, also based on Tukey’s post hoc tests. A total of 120 pots were used in this experiment, with 60 *P. australis* and 60 *B. planiculmis* plants, one per pot.

In *P. australis*, the interaction between saline–alkaline concentration and water level significantly affected all functional traits except specific leaf area and root length (Table S1). In *B. planiculmis*, the interaction significantly affected all functional traits except specific leaf area, root length, and total biomass (Table S1). For both species, the combination of drought water level (−10 cm) and medium-to-high saline–alkaline concentration treatments had significant negative effects on leaf area, ramet number, and clonal biomass (Figure 5B,D,E and Figure 6B,D,E). The interaction between flooding water level (30 cm for *P. australis* and 20 cm for *B. planiculmis*) and medium and high saline–alkaline concentration treatments significantly suppressed plant height, root biomass, and aboveground biomass in both species (Figure 5A,C,F and Figure 6A,C,F). The combination of either drought water level (−10 cm) or inundation water level (30 cm) with all three saline–alkaline concentration treatments significantly increased the below/aboveground biomass ratio of *P. australis* (Figure 5G). In contrast, the interaction between drought water level (−10 cm) or inundation water level (20 cm) and medium-to-high saline–alkaline concentration treatments inhibited the clonal biomass/belowground biomass ratios in *B. planiculmis* (Figure 6H). Overall, both drought and flooding water levels exacerbated the inhibitory effects of saline–alkaline concentration on both species, with flooding water level having a more pronounced inhibitory effect than drought water level.

### 2.4. Response of Key Functional Traits of P. australis and B. planiculmis to Environmental Stresses and Their Ability to Predict Plant Production Performance

**Figure 7:**
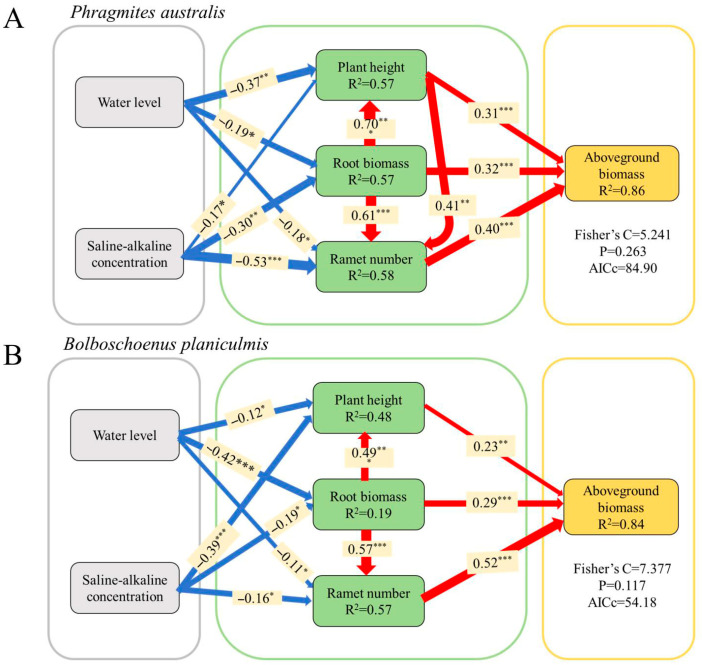
Structural equation modeling illustrating the relative impacts of predictors on aboveground biomass in *P. australis* and *B. planiculmis*. (**A**) *P. australis* and (**B**) *B. planiculmis*. The width of the arrows indicates the strength of the association between variables. Numbers on the arrows are standardized path coefficients. * *p* < 0.05; ** *p* < 0.01; *** *p* < 0.001. The r^2^ values for each response variable indicate the proportion of variance explained by the predictor. Goodness-of-fit statistics for the structural equation model are shown on the right-hand side of the model. A total of 120 pots were used in this experiment, with 60 *P. australis* and 60 *B. planiculmis* plants, one per pot.

A structural equation model (SEM) was constructed based on the results of principal component analysis (Figure 1A,B) to further examine the effects of water level and saline–alkaline concentration on the functional traits of *P. australis* and *B. planiculmis* as well as the relationships between these traits. The best-fit structural equations revealed that the three key functional traits collectively explained 86% and 84% of the variation in *P. australis* and *B. planiculmis*, respectively (Figure 7A,B). Among these traits, ramet number had the greatest direct positive effect on the aboveground biomass in both species (r = 0.40 for *P. australis*; r = 0.52 for *B. planiculmis*), while plant height and root biomass had relatively weaker and similar effects on aboveground biomass (r = 0.31 and r = 0.32 for *P. australis*; r = 0.23 and r = 0.29 for *B. planiculmis*) (Figure 7A,B). Both water level and saline–alkaline concentration negatively affected plant height, root biomass, and ramet number in both species (Figure 7A,B). Overall, saline–alkaline concentration exerted a greater influence on the functional traits of both species than water level (Figure 7A,B).

## 3. Discussion

### 3.1. Response of Functional Traits to Saline–Alkaline Stress

The effect of saline–alkaline concentration on plant functional traits depends on the species-specific characteristics. For *P. australis*, exposure to low-to-medium saline–alkaline concentration treatments significantly increased leaf area, root biomass, and clonal biomass. Previous studies have shown that some plants exhibit adaptive mechanisms to saline and alkaline environments, which can promote plant growth under low-to-moderate stress intensities [30]. The present study is consistent with the results of these previous studies. The observed increase in leaf area under low-to-medium saline–alkaline concentration treatments is consistent with the work of Xu et al. [31]. This response may be attributed to the increased leaf area, a key trait for light acquisition, which enhances resource acquisition efficiency in stressful environments, thereby enhancing photosynthetic capacity and nutrient uptake [32]. It is important to note that increased leaf area is often associated with changes in structural traits, such as leaf thickness and tissue density. The authors of future studies should further explore this relationship when investigating morphological changes in response to stress. The increase in root biomass and clonal biomass may reflect the plant’s strategy to avoid stress-induced damage by assimilating additional nutrients and water to sustain growth [33]. Our results suggest that suitable saline–alkaline concentrations can induce morphological changes and stimulate dry matter accumulation in wetland plants, thus enhancing their resilience to environmental fluctuations. Similar to findings from previous studies on the response of wetland plants such as *P. australis* to saline–alkaline stress [34,35,36], mild saline–alkaline stress may induce osmotic adjustment, allowing plants to maintain cell turgor and facilitate water uptake, thereby temporarily enhancing metabolic activity and growth. Low-level stress can also activate antioxidant defenses, protecting cells from oxidative damage and preserving redox balance. Furthermore, the upregulation of ion transporters, such as Na^+^/H^+^ antiporters, may assist *P. australis* in sustaining ionic homeostasis and metabolic function under moderate stress [37]. In our study, we also observed a reduction in plant height, ramet number, and aboveground biomass at high saline–alkaline concentrations. This reduction may result from the detrimental effects of intense saline–alkaline stress. Overall, increasing leaf area can enhance resource acquisition efficiency, promoting plant growth under low-to-moderate salinity stresses. However, high saline–alkaline stresses can disrupt the physiological processes of plants through inducing osmotic stress and ionic toxicity. These stresses impair the ability of plants to efficiently absorb nutrients and water, reduce photosynthetic capacity, and ultimately inhibit plant growth [38].

For *B. planiculmis*, exposure to low-to-medium–high saline–alkaline concentrations significantly reduced leaf area, a result consistent with the findings of Grewell et al.’s study on the effects of salinity on *Iris pseudacorus* [39]. This reduction may be attributed to the species’ relatively low saline–alkaline tolerance, leading to the inhibition of its photosynthetic capacity under saline–alkaline stresses. High saline–alkaline concentration significantly reduced both root and clonal biomass of *B. planiculmis*, and similar inhibitory effects on the belowground biomass of clonal plants, as well as the clonal/belowground biomass ratio, have also been observed in other studies [40]. The decrease in root and clonal biomass may stem from enhanced inhibition of the belowground components of plants under saline conditions, which limits root nutrient uptake and utilization [41]. This also leads to reduced rhizome extension and the inhibition of tissue division for bulb formation [40]. The observed decrease in the clonal/belowground biomass ratio may reflect the plant’s adaptive response to saline–alkaline stress, wherein it allocates more resources to roots to enhance nutrient uptake and reduce stress level [42]. This shift in biomass allocation suggests that asexual reproduction was inhibited by saline–alkaline stress, with increased root allocation reflecting a transition from clonal to rhizosphere-based reproduction.

Moreover, the increased belowground to aboveground biomass ratio under high saline–alkaline concentrations suggests that the plant allocates more resources to its belowground organs to maintain population stability in stressful environments. This strategy enhances resource utilization and improves competitiveness. These findings align with the work of Assaeed et al. [42], who explored the plasticity of *Aeluropus lagopoides* traits in brackish water habitats in response to habitat changes. Generally, biomass allocation among plant organs reflects trade-offs between above- and belowground resource acquisition [14,43]. The different responses of *P. australis* and *B. planiculmis* to saline–alkaline stress are likely linked to their respective evolutionary histories and ecological strategies. *P. australis* is widely distributed, exhibiting high genetic diversity and phenotypic plasticity, which have enabled it to adapt to variable environmental conditions over an extended evolutionary period. This adaptability contributes to its widespread distribution. In contrast, *B. planiculmis* has a more restricted distribution and shows lower plasticity, which may constrain its capacity to respond to environmental changes [18,35]. Regular monitoring of the functional characteristics of these plants can provide valuable insights into wetland health and inform the adaptation of management strategies.

### 3.2. Response of Functional Traits to the Interactions Between Water Level and Saline–Alkaline Stress

The synergistic effects of water and saline–alkaline stress on plant functional traits are largely influenced by water level and species-specific characteristics. In our study, drought conditions significantly exacerbated the inhibitory effect of medium-to-high saline–alkaline concentration treatments on the leaf area of both plant species. This finding is consistent with the results of Wang et al. [44], who reported that saline–alkaline stress adversely affected seed germination and seedling growth in six typical wetland plants. The reduction in leaf area under drought and saline–alkaline stress may function as an escape mechanism. High concentrations of salt ions in leaves can lead to stomatal closure, and a smaller leaf area in this context minimizes water loss [32,45]. Furthermore, the combination of drought conditions and medium-to-high saline–alkaline concentration treatments also significantly reduced ramet number and clonal biomass in both species. Previous studies have shown that the combined effect of drought and salinity stress exacerbates the inhibitory effect on the reproductive capacity of asexual plant species [46]. This may be due to the inhibition of bulb or rhizome formation, as well as the impaired growth and development of meristems under combined drought and saline stress conditions [47,48].

In our study, the combination of flooding and medium-to-high saline–alkaline concentration treatments had a strong inhibitory effect on plant height and aboveground biomass in both species. This result is generally in agreement with the study of Penning et al. [49] who investigated the impacts of inundation and salinity on the low-latitude salt-marsh species *Juncus roemerianus* and *Spartina alterniflora*. The underlying mechanism may involve the negative effects of complete or partial inundation on nutrient and gas uptake, as well as their transport within the plant. The resulting hypoxia, coupled with reduced light availability, limits photosynthesis and the synthesis of essential compounds, thereby reducing plant height and hindering biomass accumulation [45,50]. Several previous studies have shown that the combination of flooding and saline–alkaline stress induces physiological stress and reduces productivity across various wetland species, significantly reducing root biomass, which is consistent with our findings [9,37]. This reduction is likely due to diminished nutrient uptake by the root system, caused by ion toxicity, osmotic stress, and the hypoxic conditions induced by flooding. Furthermore, previous studies have provided essential field validation, confirming that laboratory findings are applicable to real-world ecological scenarios [9]. Future research should integrate controlled experiments with field trials to bridge the gap between mechanistic insights and ecological applications.

For *P. australis*, the combination of drought conditions and all saline–alkaline concentration treatments significantly increased the below/aboveground biomass ratio. This finding aligns with the results of An et al.’s study [48] about the effects of surface soil moisture, salinity, and alkalinity on the population characteristics of Suaeda salsa in the Momog wetland. The shift in biomass allocation patterns may reflect an adaptive strategy of wetland plants to allocate more biomass to belowground structures, thereby enhancing nutrient acquisition [51,52], thus providing a competitive advantage in stressful environments [53]. For *B. planiculmis*, the combination of flooding and all concentration saline–alkaline treatments had an inhibitory effect on clonal/belowground biomass ratios. This effect may be linked to an increasing allocation of biomass to the roots in response to water limitation [54]. Overall, our results indicated that the main factors affecting the growth of wetland plants, such as drought and flooding combined with saline–alkaline stress, exert negative synergistic effects on the survival of *P. australis* and *B. planiculmis*, respectively. Flooding generally exerts a more detrimental effect on wetland plant growth than drought, primarily due to its more significant reduction in oxygen availability to the root zone. This suggests that morphological adjustments to anaerobic conditions may be more pronounced under flooded conditions [55]. Therefore, maintaining optimal water levels that prevent both drought-induced desiccation and prolonged flooding is essential for sustaining plant productivity and biodiversity in saline–alkaline wetlands.

### 3.3. Predictive Ability of Key Traits for Plant Performance

Our study showed that three key functional traits, representing reproduction, light, and nutrient acquisition (ramet number, plant height, and root biomass), serve as effective predictors of aboveground performance in *P. australis* and *B. planiculmis*. Specifically, plant height exerted a direct influence on aboveground biomass in both species, a finding consistent with the results of the majority of related studies. For instance, Liu et al. [56] investigated the relationship between light acquisition traits and aboveground biomass in a saline herbaceous marsh ecosystem, concluding that plant height is a critical determinant of ecosystem functioning in saline wetlands [12,56,57]. Changes in plant height may also affect light competition and canopy structure, with cascading effects on understory species and overall biodiversity. While many studies have focused on the role of the root system in driving plant production and ecosystem functions [51,58,59], relatively few have examined the connection between plant root traits and overall plant functioning. Our results, however, revealed that root biomass directly influenced the aboveground performance of both *P. australis* and *B. planiculmis* in stressful conditions (Figure 7A,B). Several previous studies have shown that root traits are closely related to plant responses to environmental stressors such as salinity and water level fluctuations [46,51,60]. As a proxy for root traits, root biomass was similarly related to the aboveground performance of the plant. In addition, root biomass is closely linked to soil stabilization and organic matter accumulation, both of which are essential for maintaining wetland structure and preventing erosion.

Interestingly, our study showed that the contribution of ramet number to plant growth surpassed that of plant height and root biomass. These results suggest that reproductive traits, particularly the number of meristems, play a crucial role in determining plant performance. This may be attributed to the significant contribution of meristematic tissues to rhizome formation and ramet germination, which subsequently influence aboveground biomass. This observation is similar to the findings of Pang et al. [61], who assessed the effects of clonal propagation on plant invasion patterns in flooded and saline environments, noting that clonal integration is an effective means to enhance invasiveness. Through tillering, plants produce clonal meristems that are interconnected through stolons and rhizomes, facilitating the exchange of water, nutrients, and other resources between individuals. This interconnectedness accelerates their adaptive capacity to environmental stressors. Additionally, clonal plants tend to form dense, patchy populations through a large number of tillers to achieve rapid occupation of space and resources [62]. The importance of clonal reproductive traits in habitat colonization and subsequent survival of plants is well documented and also highlights the predictive power of morphologically defined clonal growth traits for species abundance, underscoring that species abundance within a landscape is largely influenced by their capacities for dispersal and habitat colonization [63,64]. Similarly, ramet number reflects clonal growth capacity, which in turn influences vegetation cover and microhabitat formation.

The three functional traits (plant height, ramet number, and root biomass) serve as reliable indicators of growth performance for *P. australis* and *B. planiculmis* in environments characterized by water and saline–alkaline stress. Moreover, these traits have been shown in other studies to respond predictably to variations in hydrology, salinity, and nutrient availability, effectively predicting the performance of wetland plants [29,31,65]. Understanding the changing patterns of key functional traits in *P. australis*, an emergent macrophyte (50–300 cm in height), and *B. planiculmis*, a small sedge species (20–60 cm in height), both of which are dominant herbaceous species in marsh ecosystems [66], can provide valuable insights into how ecosystem functions (e.g., aboveground biomass) in herbaceous marshes respond to environmental changes. Therefore, plant height, ramet number, and root biomass can be integrated into a strategic framework, termed RHR strategies (ranked by their importance to plant production), which can facilitate the prediction of growth performance in wetland perennial plants under the combined effects of water disturbance and saline–alkaline stress [67]. In this study, we found that the RHR strategy under saline–alkaline and water stress was characterized by a reduction in ramet number, root biomass, and plant height, which is consistent with a shift toward the conservative end of the phytoeconomic spectrum (PES). This suggests that wetland plants tend to adopt resource-conserving strategies in response to environmental stresses. This framework offers a promising tool for forecasting plant productivity in saline wetlands under future environmental changes. This strategy can guide species selection and planting design in degraded wetlands, with a functional trait-based approach supporting adaptive management and informing decisions such as water level regulation and nutrient supplementation. Monitoring changes in these traits over time can also serve as an early indicator of plant stress and ecosystem recovery processes. Current PCA methods may not fully capture the multidimensional variation in functional traits. Future studies could explore the use of nonlinear methods to more effectively investigate potential threshold effects and trait interactions. Ecological systems are typically influenced by numerous unmeasured factors, and the exclusion of important latent variables or reliance on a priori hypothesized pathways may lead to an oversimplified representation of underlying ecological processes. Therefore, while SEM provides valuable insights into the relationships between key functional traits, future studies should aim to incorporate a more comprehensive set of variables to assess the robustness of the observed relationships.

## 4. Materials and Methods

### 4.1. Study Area

The Momog National Nature Reserve (MNNR), located between 123°27′ to 124°4′ E and 45°42′ to 46°18′ N, is situated in the western region of the Songnen Plain. The total area of the reserve is approximately 144,000 ha, and it is characterized by a semi-arid temperate continental monsoon climate. Historically, the Momog wetland was characterized by high species diversity [68]. However, recent large-scale agricultural activities and water conservancy projects have significantly impacted the local environment [6,18]. These disturbances have led to increased soil salinization and noticeable degradation of wetland vegetation. The topography of this region is predominantly flat, with an average elevation of 142 m. The area experiences an average annual temperature of 4.2 °C and an average annual precipitation of 392 mm. The annual evapotranspiration rate reaches up to 1500 mm, with precipitation being concentrated between June and August, accounting for 76.6% of the total annual rainfall. This period coincides with the warm season, and seasonal fluctuations in wetland soil moisture are pronounced. The reserve is home to six main soil types: swamp soil, meadow soil, alkaline soil, black calcium soil, wind-blown sandy soil, and alluvial soil. Prominent plant species include *P. australi*, *B. planiculmis*, and *Suaeda glauca*, which are adapted to the saline–alkaline conditions prevalent in the area [3,25,69].

### 4.2. Study Species

Two dominant plant species, *P. australis* and *B. planiculmis*, were selected from the saline marsh wetland of Momog for this study. These two species are widely distributed across East Asia, Central Asia, Central Europe, and North America [24]. Data from a three-year consecutive vegetation survey indicated that the natural flooding level in the distribution area of *P. australis* in the Momog wetland ranged from 0 to 75 cm, with an average of 30 cm. In contrast, the natural flooding level in the distribution area of *B. planiculmis* ranged from 0 to 35 cm, with an average of 15 cm [18,67].

### 4.3. Sample Collection and Planting

In May 2023, rhizomes of *P. australis* and tubers of *B. planiculmis* were collected from a single soil type within the Momog National Nature Reserve. The collected *P. australis* rhizomes were cleaned, with secondary roots removed, and then cut into 18 cm segments, each containing four active bud points. The *B. planiculmis* tubers were cleaned, and the rhizomes connecting the inter-tuber sections were separated to yield individual tubers. Both the rhizomes and tubers were placed in small plastic pots (L × W × H: 35.5 cm × 25.3 cm × 11.8 cm) filled with 5 cm of moist river sand to facilitate seedling establishment. These plastic pots were incubated in a greenhouse for about two weeks with light and temperature conditions set at 12/12 h and 15/25 °C (day/night), respectively, and humidity maintained at 80%. This setup resulted in the successful establishment of about 200 *P. australis* and 200 *B. planiculmis* seedlings. The morphologically uniform *P. australis* seedlings (3~5 leaves, 18~20 cm in height) were then transplanted into plastic seedling pots (D × H: 25.5 cm × 25.3 cm), each filled with 20 cm of ordinary river sand, with 1 plant per pot. Similarly, *B. planiculmis* seedlings (10~12 cm in height) were transplanted into plastic buckets (diameter × height: 12 cm × 16 cm) filled with 15 cm of ordinary river sand, with one plant per bucket. The potted plants were placed under a shelter outside the greenhouse and watered daily to maintain moisture in the surface layer of the river sand, and the experiment began one week after transplanting, following a period of seedling retardation, depending on the condition of the seedlings.

### 4.4. Experimental Design

**Figure 8:**
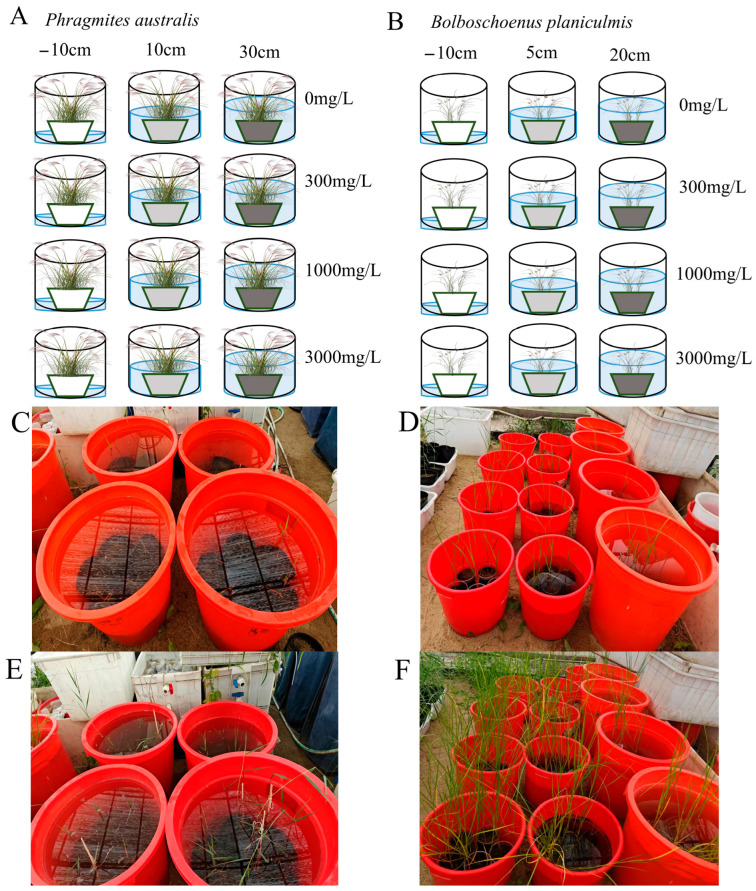
Schematic diagram of the experimental design. (**A**) *P. australis*; (**B**) *B. planiculmis*; (**C**) *P. australis* experiment beginning; (**D**) *B. planiculmis* experiment beginning; (**E**) *P. australis* before harvesting; (**F**) *B. planiculmis* before harvesting. The horizontal direction represents the water level, while the vertical direction represents the saline–alkaline concentration. The potted plants were placed under a shelter outside the greenhouse, exposed to full sunlight and ambient temperature, with five replicates per treatment. A total of 120 pots were used in this experiment, with 60 pots containing *P. australis* and 60 pots containing *B. planiculmis*, one plant per pot.

A two-factor interaction experiment design was employed, consisting of three water levels and four saline–alkaline concentrations. For *P. australis*, the three water levels were set at −10, 10, and 30 cm, while the four saline–alkaline concentrations were 0, 300 (Low), 1000 (Medium), and 3000 (High) mg/L. For *B. planiculmis*, the three water levels were −10, 5, and 20 cm, and the four saline–alkaline concentrations were 0, 300 (Low), 1000 (Medium), and 3000 (High) mg/L. The selected water levels and saline–alkaline concentrations were based on previous field studies and the characteristics of the water bodies at the sampling sites [18,70,71], aiming to simulate local water quality conditions. Field studies have shown that a water salinity of 1.0 g/L represents the threshold at which general aquatic vegetation begins to experience salinity-induced stress in the Momog National Nature Reserve, while a salinity of 4.0 g/L is the threshold at which most aquatic vegetation is effectively killed. At the collection sites for the rhizomes of *P. australis* and the tubers of *B. planiculmis* in this study, the water salinity was approximately 1500 mg/L. The saline–alkaline solution was prepared using NaCl and NaHCO_3_ in a mass ratio of 1:3. For each plant species, twelve two-factor treatment combinations were designed, with five replicates per treatment. A total of 120 pots were used in this experiment, with 60 *P. australis* and 60 *B. planiculmis* plants, one per pot. Seedlings of *P. australis* and *B. planiculmis* were irrigated to the desired water level using Hoagland half-strength nutrient solution enriched with varying concentrations of NaHCO_3_ and NaCl. The nutrient solution had the following initial composition: 0.5 mM NH_4_H_2_PO_4_, 3 mM KNO_3_, 2 mM Ca(NO_3_)_2_∙4H_2_O, 1 mM MgSO_4_∙7H_2_O, 23.13 μM H_3_BO_3_, 4.57 μM MnCl_2_∙4H_2_O, 0.382 μM ZnSO_4_∙7H_2_O, 0.16 μM CuSO_4_∙5H_2_O, 0.0695 μM MoO_3_, and 9 μM Fe-EDTA. The nutrient solution was replaced every seven days. Throughout the experimental period, water was replenished every two days to maintain the required water level for each treatment. After 10 weeks of seedling growth, the plants were harvested, and samples were collected for further analysis and index determination (Figure 8A–F).

### 4.5. Functional Trait Measurements

Following plant harvest, plant samples were separated into aboveground parts (including leaves and stems) and belowground parts (including roots and clonal structures). The belowground parts were carefully excavated during harvesting, with residual sand removed to maintain the structural integrity of the samples. The belowground samples were then divided into roots and clonal structures. Various functional traits were measured, including light acquisition traits (plant height, leaf area, and specific leaf area), nutrient and water acquisition traits (root length and root biomass), clonal traits (ramet number, total rhizome length, and clonal biomass), and biomass accumulation and allocation (aboveground biomass, belowground biomass, below/aboveground biomass ratio, clonal/belowground biomass ratio, and total biomass). Biomass was determined by drying the samples in an oven at 70 °C until a constant weight was achieved. Standardized procedures were followed for all functional trait measurements.

Plant height (PH, cm) was measured as the distance from the basal stem to the natural crown of the plant. Leaf traits were measured immediately after collection. Leaf area (LA, cm^2^) was calculated as the average area of 5–6 fully developed fresh leaves, which were scanned and analyzed using WinFOLIA software v2021 (Regent Instruments, Quebec City, QC, Canada). The leaves were then dried at 70 °C for 48 h to obtain their dry weight. Specific leaf area (SLA, cm^2^g^−1^) was calculated as the ratio of leaf area to leaf dry weight. Root length (RL, cm) was determined by scanning five randomly selected fine roots (diameter < 2 mm) and analyzing the images using WinFOLIA software. Root biomass (RB, g), clonal biomass (CB, g), aboveground biomass (AB, g), belowground biomass (BB, g), and total biomass (TB, g) were quantified by directly weighing the respective plant parts. Root biomass was determined by drying root samples in an oven at 70 °C for 48 h, followed by weighing. Clonal biomass included the rhizomes of *P. australis* and both rhizomes and tubers of *B. planiculmis*, with the dry weight representing the clonal biomass. Aboveground biomass was obtained by accumulating the stem and leaf biomass, while belowground biomass consisted of root and clonal organ biomass. The total biomass was calculated as the sum of aboveground and belowground biomass. Ramet number (RN) was recorded as the total number of ramets per pot. The total root length (TRL, cm) was calculated by measuring the length of each rhizome. The below/aboveground biomass ratio (BAR) was defined as the ratio of belowground biomass to aboveground biomass, and the clonal/belowground biomass ratio (CBR) was the ratio of clonal biomass to belowground biomass.

### 4.6. Statistical Analyses

A two-way analysis of variance (ANOVA) was conducted to examine the effects of water level and saline–alkaline concentration on plant height, ramet number, root biomass, aboveground biomass, below/aboveground biomass ratio, and clonal/belowground biomass ratio for both species. Prior to conducting the ANOVA, the normality and homogeneity of variances of the data were assessed using the Shapiro–Wilk test and Levene’s test, respectively. If necessary, the data were log-transformed to meet the assumptions of normality. In the ANOVA, saline–alkaline concentration and water level were treated as fixed factors. Tukey’s post hoc tests were then conducted to identify significant differences between treatments for variables exhibiting significant effects. A significance level of *p* < 0.05 was used to determine statistical significance in this study.

Principal component analysis (PCA) was conducted to identify the key functional traits of the two plant species based on the first principal component (PC1) axis. Single-variable linear regression models were used to explore the relationships between key functional traits and other functional traits in these two plant species. Structural equation modeling (SEM) was applied to explore the relative effect of each predictor on aboveground biomass. In the SEM analysis, saline–alkaline concentration and water level were treated as exogenous variables, plant functional traits were treated as endogenous variables, and aboveground biomass was treated as the final response variable. Models demonstrating adequate fit (*p* > 0.05) were considered to be candidate models. Model performance was evaluated using Shipley’s test of d-separation, Fisher’s C statistic, and Akaike Information Criterion corrected (AICc). The model with the lowest Fisher’s C and AICc values was selected as the most suitable model. All statistical analyses were performed using the R software package version 3.6.3, with structural equation modeling carried out using the “piecewiseSEM” package [72].

## 5. Conclusions

This study investigated the responses of the functional traits of two saline marsh herbaceous plants, *P. australis* and *B. planiculmis*, to varying saline–alkaline concentrations and water levels. The results demonstrated that the effects of low and medium saline–alkaline concentrations on these two plant species were species-dependent, while high saline–alkaline concentrations significantly inhibited their growth. Among the two species, *P. australis* exhibited greater saline–alkaline tolerance compared to *B. planiculmis*. Both drought and flooding exacerbated the negative effects of saline–alkaline stress on the plant growth of both species, with flooding having a more pronounced inhibitory effect than drought on *P. australis* and *B. planiculmis*. Among the plant functional traits examined, plant height, root biomass, and ramet number exhibited the most significant influence on overall plant performance, with the ramet number contributing more than the other two functional traits. Based on these findings, we conclude that the RHR strategy may serve as an effective adaptive strategy for saline marsh herbaceous plants in response to variations in saline–alkaline concentration and water level fluctuations. Moreover, it is essential to focus on functional traits related to reproductive capacity when assessing the response of wetland plants to environmental stressors. Theoretically, this study aimed to elucidate the ecological adaptation mechanism of *P. australis* and *B. planiculmis* in response to environmental stress. Practically, it provides valuable insights for the population restoration of these species, the maintenance and protection of ecosystem stability, and the restoration and management of degraded saline–alkaline wetland ecosystems. However, these results were obtained under short-term controlled conditions and may not fully capture the complexity of natural environments. In the field, plants are exposed to fluctuating stress levels, interspecific competition, and varying seasonal conditions. Future studies should incorporate additional physiological and ecological indicators, especially those related to reproductive capacity, as well as conduct long-term, cross-seasonal field experiments in order to achieve a more comprehensive and objective understanding of the adaptive mechanisms of wetland plants under environmental stresses.

## Data Availability

Data are contained within the article and Supplementary Materials.

## Supplementary Materials

The following supporting information can be downloaded at: https://www.mdpi.com/article/10.3390/plants14142112/s1. Table S1: Two-factor analysis of variance (ANOVA) on the effects of water level (W) and saline–alkaline concentration (S) on the functional traits of *P. australis* and *B. planiculmis*.
