# Supplementary material for: The Response of the Functional Traits of Phragmites australis and Bolboschoenus planiculmis to Water and Saline–Alkaline Stresses
Source: Plants (Basel). 2025 Jul 9;14(14):2112. doi: 10.3390/plants14142112 (PMC12299409; doi:10.3390/plants14142112)
Supplement: Supplementary file 1 [file plants-14-02112-s001.zip › plants-3649304-supplementary.pdf]

Table S1. Two-factor analysis of variance (ANOVA) on the effects of water level (W) and saline-alkaline concentration (S) on functional traits of *P. australis* and *B. planiculmis*

Variables	Factors	*P.australis*			*B.planiculmis*		
		df	*F*	*P*	df	*F*	*P*
Plant height (cm)	W	2	12.985	**<0.001**	2	11.131	**<0.001**
	S	3	6.474	**0.001**	3	9.272	**<0.001**
	W×S	6	2.089	**0.012**	6	3.597	**0.005**
Leaf area (cm^2)	W	2	38.958	**<0.001**	2	19.557	**<0.001**
	S	3	12.544	**<0.001**	3	15.831	**<0.001**
	W×S	6	3.862	**0.003**	6	2.223	**0.047**
Specific leaf area (cm^2g^{-1})	W	2	7.795	**0.001**	2	3.802	**0.029**
	S	3	3.493	**0.023**	3	0.213	0.887
	W×S	6	1.447	0.217	6	0.745	0.617
Root length (cm)	W	2	1.779	0.18	2	5.956	**0.005**
	S	3	2.157	0.105	3	3.583	**0.02**
	W×S	6	3.046	**0.013**	6	1.21	0.318
Root biomass (g)	W	2	38.314	**<0.001**	2	14.869	**<0.001**
	S	3	4.974	**0.004**	3	0.575	**0.034**
	W×S	6	1.599	**0.018**	6	1.392	**0.037**
Ramet number	W	2	21.966	**<0.001**	2	35.608	**<0.001**
	S	3	3.047	**0.038**	3	3.269	**0.029**
	W×S	6	4.942	**0.001**	6	3.784	**0.004**
Total rhizome length (cm)	W	2	61.402	**<0.001**	2	15.585	**<0.001**
	S	3	34.465	**<0.001**	3	1.87	0.147
	W×S	6	7.561	**<0.001**	6	2.419	**0.04**
Clonal biomass (g)	W	2	60.935	**<0.001**	2	13.953	**<0.001**
	S	3	8.143	**<0.001**	3	4.796	**0.005**
	W×S	6	2.67	**0.026**	6	2.145	**0.025**
Aboveground biomass (g)	W	2	25.487	**<0.001**	2	24.447	**<0.001**
	S	3	8.175	**<0.001**	3	4.843	**0.005**
	W×S	6	2.8	**0.02**	6	3.242	**0.009**
Belowground biomass (g)	W	2	53.3	**<0.001**	2	14.205	**<0.001**
	S	3	6.986	**0.001**	3	4.455	**0.008**
	W×S	6	2.357	**0.045**	6	2.061	**0.036**
Belowground/aboveground biomass ratio	W	2	53.373	**<0.001**	2	2.419	0.1
	S	3	0.377	**0.007**	3	1.852	**0.015**
	W×S	6	1.591	**0.017**	6	4.711	**0.001**
Clonal/belowground biomass ratio	W	2	27.065	**<0.001**	2	14.173	**<0.001**
	S	3	6.513	**0.001**	3	2.004	0.126
	W×S	6	2.882	**0.018**	6	3.401	**0.007**
Total biomass (g)	W	2	38.559	**<0.001**	2	18.79	**<0.001**
	S	3	7.919	**<0.001**	3	5.286	**0.003**
	W×S	6	2.469	**0.037**	6	2.117	0.068
